# Radiation-Induced Cardiotoxicity in Hypertensive Salt-Sensitive Rats: A Feasibility Study

**DOI:** 10.3390/life15060862

**Published:** 2025-05-27

**Authors:** Dayeong An, Alison Kriegel, Suresh Kumar, Heather Himburg, Brian Fish, Slade Klawikowski, Daniel Rowe, Marek Lenarczyk, John Baker, El-Sayed Ibrahim

**Affiliations:** 1 Department of Biomedical Engineering, Medical College of Wisconsin, Milwaukee, WI 53226, USA; dan@mcw.edu; 2 Deaprtment of Physiology, Medical College of Wisconsin, Milwaukee, WI 53226, USA; 3 Department of Pathology & Laboratory Medicine, Medical College of Wisconsin, Milwaukee, WI 53226, USA; 4 Department of Radiation Oncology, Medical College of Wisconsin, Milwaukee, WI 53226, USA; 5 Department of Mathematical and Statistical Sciences, Marquette University, Milwaukee, WI 53233, USA; daniel.rowe@marquette.edu; 6 Department of Surgery, Medical College of Wisconsin, Milwaukee, WI 53226, USAjbaker@mcw.edu (J.B.); 7 Department of Radiology, Medical College of Wisconsin, Milwaukee, WI 53226, USA

**Keywords:** cardiac MRI, hypertension, cardiotoxicity, radiation therapy, myocardial strain

## Abstract

Radiation therapy (RT) plays a vital role in managing thoracic cancers, though it can lead to adverse effects, including significant cardiotoxicity. Understanding the risk factors like hypertension in RT is important for patient prognosis and management. A Dahl salt-sensitive (SS) female rat model was used to study hypertension effect on RT-induced cardiotoxicity. Rats were fed a high-salt diet to induce hypertension and then divided into RT and sham groups. The RT group received 24 Gy of whole-heart irradiation. Cardiac function was evaluated using MRI and blood pressure measurements at baseline, 8 weeks and 12 weeks post-RT. Histological examination was performed after the last timepoint or animal death. The hypertensive RT rats demonstrated significant decreases in left-ventricular ejection fraction (EF) (45 ± 7.2%) compared to sham (68 ± 7.3%). Furthermore, circumferential (Ecc) and radial (Err) myocardial strains were significantly reduced (Ecc: −7.4 ± 2.0% RT rats vs. −11 ± 2.4% sham; Err: 15 ± 6.5% RT rats vs. 23 ± 8.9% sham). Histological analysis revealed significant pathophysiological remodeling post-RT, including nuclear size, interstitial fibrosis, necrosis, and the presence of inflammatory cells. This study provides valuable insights into the cardiotoxic effects of RT in the context of hypertension, highlighting the potential of using MRI for improved risk assessment with potential for future clinical translation.

## 1. Introduction

The increasing prevalence of cancer and advancement in treatment methods have led to a growing number of patients receiving radiation therapy (RT) [1,2,3]. Specifically, lung cancer patients commonly undergo RT as a crucial part of their treatment plan, which has been proven to enhance local control and survival [2,4,5,6]. Despite treatment advancements improving survival, the radiation effect on the heart remains a major concern [2,7,8]. As cardiac dysfunction can progress to heart failure if not appropriately managed [2,3,5,9,10], the identification of sensitive, non-invasive biomarkers for the early detection of subclinical cardiac dysfunction is crucial to reduce the morbidity and mortality associated with thoracic RT.

RT-induced cardiotoxicity can manifest in various forms, ranging from mild to severe, including but not limited to pericarditis [6,11,12,13], coronary artery disease [6,13,14,15,16], valvular heart disease [6,13,17], and myocardial dysfunction [6,13,18,19,20]. While numerous studies have focused on the effects of radiation dose and the volume of the heart exposed to radiation on the development of cardiotoxicity [2,7,21,22], the effect of major baseline risk factors on RT-induced cardiotoxicity is not fully elucidated. Specifically, hypertension is a prevalent and well-established risk factor in lung cancer patients [23,24,25,26]. Recent evidence suggests that hypertension may play a significant role in the development of RT-induced cardiotoxicity [21,27,28,29,30]. Hypertensive patients may have an increased vulnerability to RT-induced damage to the heart, potentially exacerbating the risk of cardiotoxicity [1,2,21,23,25].

We have previously studied the effect of RT on normotensive rats [31]. In this study, we further investigate the incremental effect of hypertension on RT-induced cardiotoxicity in the same animal model. The pilot results from this study have the potential studies on humans towards enhancing the management of cardiotoxicity risks and improving outcomes in cancer patients.

## 2. Materials and Methods

### 2.1. Animal Model and Irradiation Procedure

**Figure 1:**
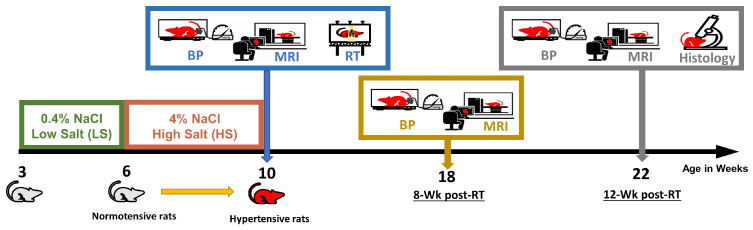
SS rats were fed high-salt diet for 4 weeks to develop hypertension before RT/sham treatment. Blood pressure measurements and MRI scans were conducted at baseline as well as 8- and 12-weeks post-RT or sham treatment. Histology analyses were conducted after last experiment or animal death.

The study (Figure 1) was approved by the institutional animal care committee of the Medical College of Wisconsin under protocol number AUA996. Dahl salt-sensitive (SS) rats, which have been extensively used in the investigation of hypertension and cardiac complications [32,33,34,35], were used in this study. Inbred SS female rats (n = 6) were administered a standard low-salt diet (0.4% NaCl) from 3 to 6 weeks of age [36]. To induce hypertension, the rats were fed high-salt diet (4% NaCl) from 6 to 10 weeks of age, after which they resumed a low-salt diet [32,33,37,38].

**Figure 2:**
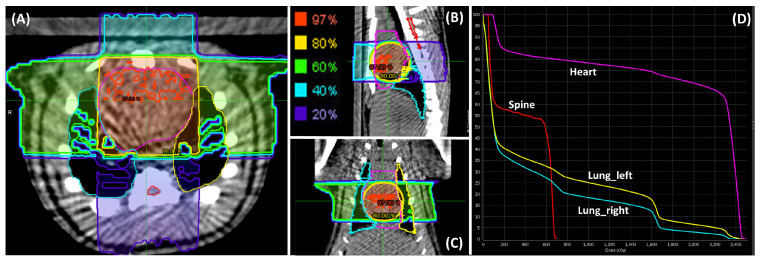
Dose distribution in different organs. Contours of lungs (right and left), heart, and spine visualized using MIM software in (**A**) transverse, (**B**) sagittal, and (**C**) coronal views. Regions receiving 97% of the prescribed dose were displayed in red, 80% in yellow, 60% in green, 40% in cyan, and 20% in purple. The dose volume histogram (DVH) is presented in (**D**).

At 10 weeks of age, the rats were randomly allocated to two groups: an RT group (n = 4) and a sham non-irraidated group (n = 2). The RT group underwent whole-heart irradiation with a dose of 24 Gy, guided by onboard cone beam computed tomography for precise targeting (one anterior-posterior beam and two lateral beams, 225 kVp, 13 mA, and clockwise gantry rotation direction) [39]. The dose rate was 2.72 Gy/min, administered using high-percision image-guided X-RAD SmART irradiator (Precision X-Ray, North Brandford, CT, USA). Figure 2 shows dose distribution in different organs. The radiation dose of 24 Gy was selected based on previous studies showing that single fraction doses in the range of 20–30 Gy reliably produce measurable cardiac dysfunction and fibrotic remodeling in rodent models [39]. The use of a single fraction high dose allows us to study early-stage cardiac responses while minimizing variability introduced by fractionation.

Blood pressure, including systolic blood pressure (SBP), diastolic blood pressure (DBP), and pulse, was measured using a tail-cuff Visitech system at the same timepoints of the MRI scans.

### 2.2. MRI Scans and Image Analysis

The rats were imaged on a small-animal 9.4T MRI scanner (Bruker, Rheinstetten, Germany). The MRI scan included both cine and tagged images acquired [40,41] along with a full stack of short-axis (SAX) and long-axis (LAX) cine slices covering the whole left ventricle (LV). Three SAX-tagged slices (basal, mid-ventricular, and apical) were acquired in addition to LAX slices. The cine sequence imaging parameters were as follows: repetition time (TR) = 6.25 ms, echo time (TE) = 2.2 ms, flip angle = 15°, matrix = 176 × 176, slice thickness = 1 mm, bandwidth = 526 Hz/pixel, scan time 2 min/slice. The tagging sequence imaging parameters were similar to cine imaging, except for the following: TE = 2.5 ms, matrix = 256 × 256, bandwidth = 375 Hz/pixel, scan time = 4–5 min/slice [40].

Cine image processing was conducted using the cvi42 software (Version 5) (Circle Cardiovascular Imaging, Calgary, AB, Canada). The measurements from all the SAX slices were used to evaluate global cardiac functions including end-diastolic volume (EDV), end-systolic volume (ESV), stroke volume (SV), ejection fraction (EF), and mass.

The tagged images were analyzed using the sinusoidal modelling technique [42,43] (InTag, Lyon, France) to measure the circumferential (Ecc), radial (Err), and longitudinal (Ell) strain. The analysis was performed on the SAX images acquired at the basal, mid-ventricular, and apical levels and on the LAX images. Strain analysis was repeated by the same observer (>2 months) and by another observer to assess data reliability.

### 2.3. Histological Analysis

The hearts were harvested from fully anesthetized RT and sham rats at 12 weeks post-RT or at the time of death. The isolated hearts were handled using standard procedures [44]. Fixed tissue samples were embedded in paraffin with sections taken at SAX levels from the basal, mid-ventricular, and apical regions of the LV. Four-micrometer sections were cut from each block and stained with hematoxylin and eosin (H&E) and Masson’s trichrome, according to standard methods. Furthermore, for mast cell staining, slides were first deparaffinized using Xylene and subsequently rehydrated through descending concentrations of ethanol. Each slide was covered in a 0.1% toluidine blue in 1% sodium chloride (pH 2.0) for 3 min. Image acquisition utilized a Nikon Eclipse 50i upright microscope, with eighteen images from each level (basal, mid-ventricular, and apical) cropped for mast cell staining. Quantification was performed by counting mast cells per high-power field by a trained pathologist.

### 2.4. Statistical Analysis

Descriptive statistics were calculated for all measured variables, which include SBP, DBP, pulse, EDV, ESV, SV, EF, mass, Ecc, Err, and Ell. Data are expressed as the mean ± standard deviation (SD). Because of small sample size, a non-parametric test, a Mann–Whitney U test, was applied for all variables with *p* < 0.05 considered significant. Bland–Altman analysis [45] was conducted to assess intra-observer and inter-observer variabilities in the generated measurements.

## 3. Results

### 3.1. Physiological Results and MRI-Derived Cardiac Function

The hypertensive rats developed high blood pressure at baseline [33,34], persisting throughout the experiment (Table 1), compared to the lower blood pressure observed in low-salt diet rats [23,25,35,38,46]. At baseline, both sham and RT groups had high SBP and DBP. By 8 weeks post-RT/sham, the sham group showed increased SBP, unlike the stable RT group. At 12 weeks, the sham group’s SBP and DBP significantly rose, while the RT group’s SBP decreased and DBP declined noticeably. As for the pulse rate, the sham group demonstrated a higher pulse rate compared to the RT group. This trend persisted at 8 weeks post-RT, with the sham group showing an increase and the RT group a slight increase. Interestingly, by 12 weeks post-RT, the sham group’s pulse slightly decreased, and the RT group further decreased. All euthanasia procedures were performed in accordance with the institutional animal care and using guidelines to ensure humane endpoints.

EDV was similar for both groups at baseline. As the study progressed to 8 weeks post-RT, both groups exhibited an increase. By the 12-week post-RT evaluation, both groups maintained nearly equivalent EDV levels. ESV initially was slightly lower in the sham group compared to the RT group at baseline. By 8 weeks post-RT, the sham group had a minimal rise in ESV, but the RT group observed a decrease. By 12 weeks post-RT, the RT group exhibited a significant increase in ESV. Regarding SV, baseline measurements were close between the sham and RT groups. By 8 weeks post-RT, there was an evident increase in both groups. But at the 12-week post-RT timepoint, the patterns diverged, with the sham group maintaining its SV while the RT group showing a decrease. Baseline assessments revealed comparable EF values in the two groups. By 8 weeks post-RT, EF in the RT group and sham group increased and slightly increased, respectively. At 12 weeks post-RT, EF in the sham group decreased, whereas it significantly declined in the RT group. At baseline, both sham and RT groups had comparable mass measurements. By 8 weeks post-RT, both groups exhibited an increase in mass, with this trend continuing through 12 weeks post-RT, where the mass measurements remained closely aligned between the two groups.

**Figure 3:**
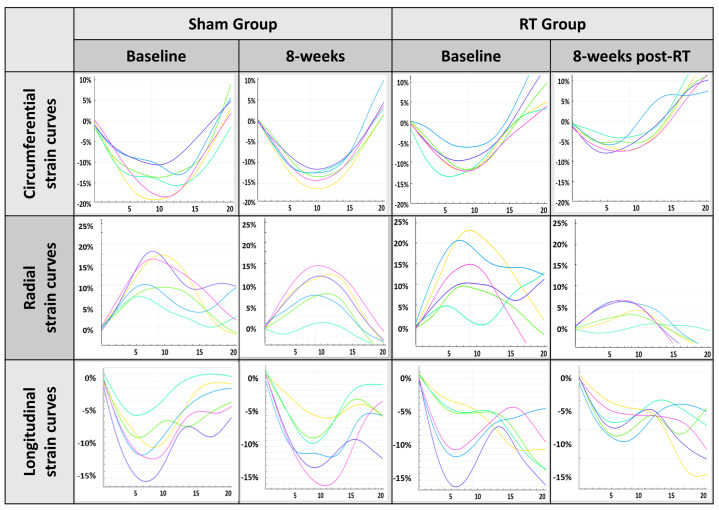
MRI−derived strain curves for the sham and RT groups show similar patterns in strain curves at baseline for circumferential, radial, and longitudinal strains. However, at 8 weeks post-RT, while the sham rats maintain comparable strain measurements, the irradiated rats show reduced strain magnitudes.

At baseline, both the sham and RT rats exhibited similar strain measurements across Ecc, Err, and Ell as depicted in Figure 3. The Ecc values for both groups were in close proximity. By 8 weeks post-RT, the sham group slightly increased, whereas the RT rats showed a sharper decrease. This disparity became more evident by the 12-week mark, with the sham group ascending further and the RT group settling. For Err, the baseline values were closely matched. This close range persisted at 8-week post-RT. Yet, by 12 weeks post-RT, both groups exhibited a rise. Lastly, Ell measurements for both the sham and RT groups were similar at baseline. By 8 weeks post-RT, a subtle decrease was noted in both groups. However, the 12-week post-RT measurement revealed a consistent trend while the sham group remained close to its previous value, but the RT group showed a noticeable drop.

**Figure 4:**
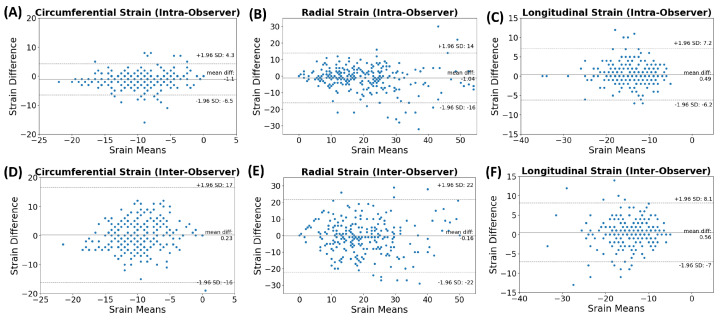
Bland−Altman analysis illustrating consistency in strain for repeated measurements across multiple trials and observers. Diagrams (**A**–**C**) signify intra−observer and (**D**–**F**) indicate inter-observer measurements, denoting circumferential (**A**,**D**), radial (**B**,**E**), and longitudinal (**C**,**F**) strains. The majority of measurement differences are located within the mean ± 2SD range, implying low variability, thereby underlining the reliability and reproducibility of these strain measurements.

To ascertain whether there was a significant difference in the distributions of the variables between the sham and RT groups, we performed the Mann–Whitney U test. Despite observing variations in the trends of physiological and MRI parameters between the groups, the Mann–Whitney U test revealed no statistically significant difference between the groups for all the variables considered. The Bland–Altman analysis revealed low intra- and inter-observer variabilities in the strain measurements (Ecc, Err, and Ell), with almost all measurement differences lying within the agreement range of mean ± 2SD of the measurement differences (Figure 4).

### 3.2. Histopathologic Results

**Figure 5:**
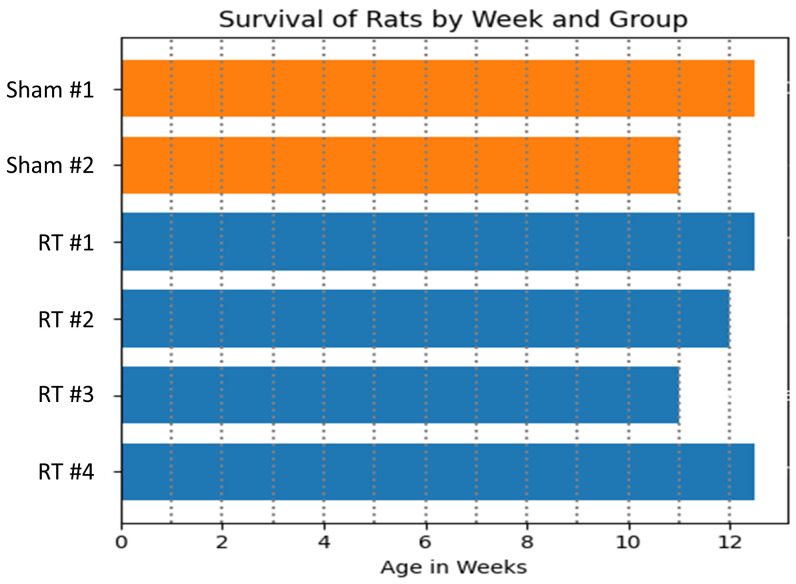
The bar chart illustrates the survival status of rats in the sham (orange) and RT (blue) groups over a 12-week post-RT period. In the sham group, one rat was euthanized following a seizure at the end of the 11th week, and another rat was euthanized by the 12th week. In the RT group, two rats developed heart failure and were euthanized, while another rat died, and one rat died during tail-cuff blood pressure measurement.

Figure 5 demonstrates survival data in the studied rats. Initially, the sham group comprised two rats, and the RT group had four rats. By 11 weeks post-RT, complications following a seizure led to the euthanasia of one rat from the sham group, and another rat from the RT group died. At 12 weeks post-RT, two rats from the RT group developed heart failure and were consequently euthanized. Another rat from the same group died during the blood pressure measurement. Finally, one sham rat was euthanized at 12 weeks post-RT.

**Figure 6:**
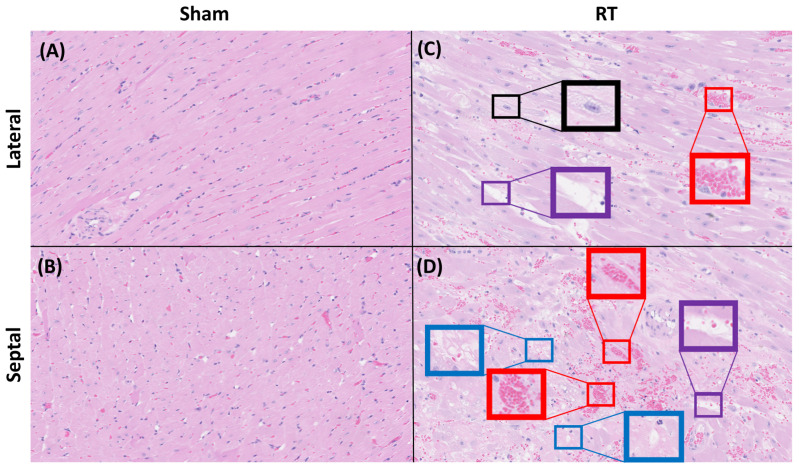
Histopathological changes in rat cardiac tissues observed using H&E staining (40× magnification). A notable difference in staining intensity is observed, with the irradiated group exhibiting a brighter pink hue, suggestive of a reduction in cytoplasmic proteins. In the sham group (**A**,**B**), normal histoarchitecture is displayed, characterized by well-organized and branched cardiac myofibers in the cardiomyocytes. In contrast, the irradiated rat (**C**,**D**) shows increased nuclear size (black boxes), interstitial fibrosis and necrosis (purple boxes), increased capillary density and presence of inflammatory cells (red boxes), as well as vacuolization of sarcoplasm (blue boxes) in (**D**). In the RT group, the presence of inflammatory cells is more prominent in the septal wall, while interstitial fibrosis and necrosis are more pronounced in the lateral wall.

**Figure 7:**
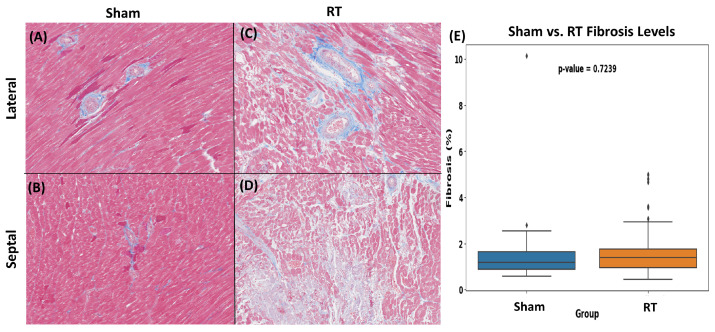
Masson’s trichrome staining (40× magnification) of rat myocardial tissue from sham (**A**,**B**) and irradiated tissues (**C**,**D**). The blue staining indicates the presence of collagen. Notably, the irradiated group shows signs of tissue damage (white spot). In (**E**), the interstitial collagen volume fraction is quantified for both sham and irradiated groups, with values expressed as mean ± SD. The difference of interstitial collagen between the two groups was not statistically significant, with a *p*-value of 0.72.

**Figure 8:**
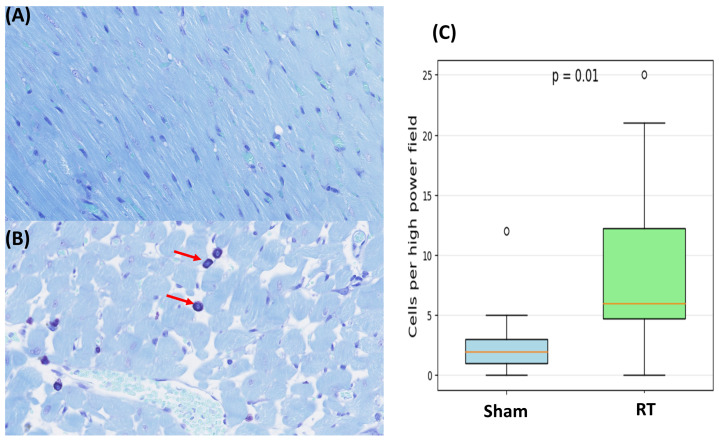
Toluidine blue staining (40× magnification) of rat myocardial tissue from sham (**A**) and irradiated groups (**B**). The dark blue staining indicates the mast cell (red arrows). (**C**) The box plot shows that there is a significant difference between two groups as *p*-value = 0.01.

Multiple fields of view from myocardial tissue sections of the sham and RT groups were examined. Specifically, for each staining method employed, 26 fields of view were analyzed for the sham group and 78 for the RT groups. These fields of view represent zoomed and cropped images of stained myocardial tissues. H&E staining demonstrated distinct differences in myocardial tissue organization between the sham and RT groups (Figure 6). The RT group exhibited a more subtle pink staining color in the H&E staining process, which could potentially signify a reduction in the concentration of cytoplasmic proteins. Compared to the sham group, which shows well-organized and normal histoarchitecture, the RT group exhibits several histopathological changes. These include increased nuclear size, interstitial fibrosis and necrosis, heightened capillary density, the presence of inflammatory cells, and sarcoplasmic vacuolation (Figure 6). RT-induced damage can manifest as a chronic process, marked by the collagen deposition and excessive production of fibrosis. The levels of fibrosis were assessed in myocardial tissue of the hypertensive rats in both sham and RT groups using Masson’s trichrome staining (Figure 7). Upon employing Masson’s trichrome staining, areas indicative of tissue damage, including areas of fibrosis, necrosis, and augmented extracellular matrix components, were detected in the myocardial tissue. The analysis revealed comparable fibrosis levels in both groups, with the sham group averaging 1.65 ± 1.86% and the RT group at 1.56 ± 0.94%. The difference in fibrosis levels between the groups was not statistically significant (*p*-value = 0.72). Additionally, the toluidine blue staining revealed a significant increase in mast cell infiltration within the LV in the RT group compared to the sham group (*p*-value < 0.05) (Figure 8).

## 4. Discussion

In this study, we investigated RT’s impact on cardiac function in hypertensive rats, utilizing MRI and histological analysis to assess alterations in cardiac parameters and pathology. The results demonstrated significant changes in cardiac function as evidenced by both histology and strain measurements. The combination of pre-existing hypertension and radiation exposure may accelerate myocardial remodeling through several interconnected mechanisms. Hypertension in Dahl SS rats induces structural stress on the myocardium, which may prime the tissue for enhanced radiation sensitivity. Radiation-induced inflammation and mast cell infiltration further contribute to myocardial damage, as reflected in the early appearance of fibrosis and vacuolization in the lateral wall. Clinically, this model may reflect high-risk cancer patients with underlying cardiovascular disease, emphasizing the importance of early detection and protective strategies. Although the high-salt diet was administered only transiently from 6 to 10 weeks of age to induce hypertension, the hypertensive phenotype persisted throughout the study period, as demonstrated by sustained elevated blood pressures at all timepoints. This is consistent with the well-established characteristics of the Dahl SS rat model, where hypertension is largely irreversible even after reverting to a low-salt diet [32,33,34,35]. Additionally, salt-induced hypertension in SS rats leads to renal injury that is not fully reversible, resulting in chronic kidney damage [32,34,37]. Renal dysfunction may independently contribute to adverse cardiac remodeling, compounding the cardiotoxic effects of RT. Thus, in this study, the persistent hypertension and potential renal injury both likely played roles in the observed cardiac dysfunction. Future investigations involving detailed assessment of renal function would help further clarify the interactions between hypertension, renal impairment, and radiation-induced cardiotoxicity. The hypertensive rats maintained elevated levels of blood pressure throughout the study. At 8 weeks post-RT, the rats showed stable levels of both SBP and DBP, while the sham group experienced an increase. Furthermore, a post-RT increase in pulse rate was observed in both RT and sham groups, although the sham group maintained a higher pulse rate.

Baseline EF values were comparable between sham and RT rats. A moderate increase in EF was seen in the sham group at 8 weeks post-RT while the RT group exhibited a pronounced increase at the same timepoint. Both Ecc and Err strains were found to decrease (in absolute value) at 8 weeks post-RT compared to the sham group. However, we noted an unexpected increase in strains at 12 weeks post-RT in the hypertensive rats. The variation in strain patterns in the hypertensive rats in this study compared to previously reported results in similar normotensive rats [41] could reflect the complex interactions between hypertensive conditions and RT. Hypertension is known to induce alterations in the myocardial structure and function [23,25,47], which might influence the cardiac response to radiation. Therefore, myocardium strain changes may be indicative of a compensatory behavior in the hypertensive rats.

The histopathological analysis using H&E staining revealed distinct differences in myocardial tissue organization between the sham and RT groups, highlighting the structural modifications effect of RT on the myocardium. Observations from Masson’s trichrome staining revealed a higher level of fibrosis in myocardial tissue upon irradiation although the differences were not statistically significant when compared to the sham group. Notably, the toluidine blue staining exhibited a statistically significant difference between two groups, indicating mast cell infiltration. Mast cells are known to be involved in inflammation and fibrosis and may play a pivotal role in mediating RT-induced cardiac changes.

**Figure 9:**
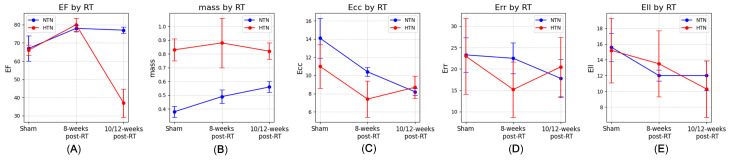
Comparative analysis of various physiological metrics post radiation therapy (RT) between normotensive (NTN) and hypertensive (HTN) rats. Each plot depicts mean ± SD for (**A**) ejection fraction (EF), (**B**) mass, (**C**) circumferential (Ecc), (**D**) radial (Err), and (**E**) longitudinal (Ell) strains at three distinct time points: sham, 8 weeks post-RT, and 10/12 weeks post-RT. Blue and red markers represent NTN and HTN rats, respectively.

In comparing irradiated hypertensive rats to previously reported irradiated normotensive rats [31] (Figure 9), significant differences in cardiac responses to RT were observed. Hypertensive rats displayed a pronounced hypertrophic response with substantially increased cardiac mass, diverging from the gradual increase seen in normotensive rats. This highlights the impact of hypertension on pathological cardiac remodeling.

Both groups initially showed an EF increase post-RT, but by 10/12 weeks post-RT, the hypertensive group experienced a substantial EF decrease, while the normotensive group sustained their elevated levels. This suggests an exacerbated vulnerability of the hypertensive myocardium to radiation.

The changes in Ecc, Err, and Ell between groups at varying stages underscore the differential myocardial response to radiation in the presence of hypertension. Especially compelling are the robust correlations among these strain parameters within the hypertensive group. Even the normotensive group’s distinct correlation between mass and Ecc could indicate a radiation-induced myocardial stress response that is hypertensive-independent.

Our findings, compared with data on normotensive rats [31,41], highlight the modulatory role of hypertension in cardiac response to radiation. The hypertensive rats exhibit hyperkinetic behavior at the third timepoint, diverging from the decreasing trend in normotensive rats, aligning with findings from the hypertensive SS rat model [37,48,49]. These comparisons underline the importance of developing tailored therapeutic strategies and risk assessments for hypertensive patients undergoing RT, considering their distinct cardiac adaptation to RT.

This study has limitations. First, the small sample size may limit the statistical power and generalizability of the results. Nevertheless, the results clearly demonstrate different contractility patterns in the hypertensive rats compared to normotensive rats, which emphasizes the incremental RT-induced cardiac damage in the presence of hypertension.

Despite a slight difference in last follow-up timepoint between the hypertensive and normotensive rats [31], our results clearly demonstrate a worse effect of baseline hypertension on cardiac function than RT alone. The time course of RT-induced cardiac damage is crucial; for instance, the decreasing strain in normotensive rats from 8 weeks to 10 weeks post-RT suggests progressive decline due to cumulative radiation effects. Conversely, the increased strain at 12 weeks post-RT in hypertensive rats might indicate potential regional hyperkinetic contractility despite reduced EF, which warrants further investigation in future larger studies.

Another limitation is this study is constrained to observations up to 12 weeks post-RT, potentially overlooking longer-term RT effects, particularly with baseline hypertension. Nevertheless, previous studies demonstrate continued deterioration in EF in this rat model which results in heart failure by the 20th week post-RT [39].

## 5. Conclusions

In conclusion, this pilot study provided valuable insights into the effects of RT on hypertensive rats, offering a more clarification of how RT might interact with hypertension to escalate cardiotoxicity. MRI findings indicate significant deterioration of myocardial contractility following RT, as demonstrated by decreased LV EF and strain measurements and confirmed by histopathological analysis. The promising results from this study underscore the damaging impact of RT on cardiac function, particularly in the presence of hypertension, which has potential translational by conducting clinical trial for better treatment management and improved outcomes in cancer patients receiving RT.

## Figures and Tables

**Table 1:** Comparison of cardiac and physiological parameters between sham and RT groups at baseline, 8 weeks, and 12 weeks post-experiment. Parameters included systolic blood pressure (SBP in mmHg), diastolic blood pressure (DBP in mmHg), pulse rate (in bpm), end-diastolic volume (EDV in mL), end-systolic volume (ESV in mL), stroke volume (SV in mL), ejection fraction (EF%), mass (in g), and circumferential, radial, and longitudinal strains (Ecc, Err, and Ell%). Values are presented as mean ± SD.

	Baseline		8 Weeks Post-RT		12 Weeks Post-RT	
	Sham	RT	Sham	RT	Sham	RT
SBP (mmHg)	232.5 ± 12.0	223 ± 37.4	280 ± 8.5	223.3 ± 38.5	306	181 ± 54.6
DBP (mmHg)	174 ± 35.4	176.3 ± 51.9	217 ± 29.7	165.3 ± 60.8	246	93.3 ± 50.1
Pulse (bpm)	433 ± 34	410 ± 33	460 ± 59	426 ± 39	414	399 ± 50
EDV (mL)	0.38 ± 0.01	0.39 ± 0.04	0.44 ± 0	0.47 ± 0.05	0.44	0.47 ± 0.07
ESV (mL)	0.14 ± 0.02	0.17 ± 0.06	0.15 ± 0.01	0.1 ± 0.02	0.2	0.31 ± 0.08
SV (mL)	0.24 ± 0.03	0.25 ± 0.04	0.29 ± 0.01	0.38 ± 0.05	0.24	0.17 ± 0.01
EF (%)	63 ± 7.1	64.5 ± 11.7	66 ± 2.8	80 ± 3.6	56	36.5 ± 7.8
Mass (g)	0.59 ± 0.01	0.61 ± 0.05	0.83 ± 0.08	0.88 ± 0.18	0.83	0.82 ± 0.06
Ecc (%)	−10.7 ± 2.7	−9.4 ± 2.3	−11 ± 2.4	−7.4 ± 2.0	−13.7 ± 3.8	−8.7 ± 1.2
Err (%)	20.3 ± 8.9	22.8 ± 7.8	23 ± 8.9	15.2 ± 6.5	28.7 ± 8.1	20.5 ± 6.9
Ell (%)	−15.9 ± 5.0	−14.5 ± 6.3	−15.2 ± 4.1	−13.5 ± 4.2	−15.8 ± 5.4	−10.3 ± 3.6

## Data Availability

Restrictions apply to the datasets.

## Abbreviations

The following abbreviations are used in this manuscript:

RT	Radiation therapy
SS	Salt-sensitive
EF	Ejection fraction
SBP	Systolic blood pressure
DBP	Diastolic blood pressure
Ecc	Circumferential strain
Err	Radial strain
Ell	Longitudinal strain
SAX	Short axis
LAX	Long axis
LV	Left ventricle
TR	Repetition time
TE	Echo time
EDV	End-diastolic volume
ESV	End-systolic volume
SV	Stroke volume
H&E	Hematoxylin and eosin
SD	Standard deviation
